# Decoding Fluid Flow Characteristics Through Distributed Acoustic Sensing: A Novel Approach

**DOI:** 10.3390/s25072011

**Published:** 2025-03-23

**Authors:** Haochu Ku, Kunpeng Zhang, Xiangge He, Min Zhang, Hailong Lu

**Affiliations:** 1 Beijing International Center for Gas Hydrate, School of Earth and Space Sciences, Peking University, Beijing 100871, China; haochuku@stu.pku.edu.cn (H.K.);; 2 State Key Laboratory of Petroleum Resources and Prospecting, China University of Petroleum, Beijing 102249, China; 3 Dongguan Institute of Optics and Electronics, Peking University, Dongguan 523000, China; 4 Institute of Ocean Research, Peking University, Beijing 100871, China

**Keywords:** distributed acoustic sensing, flow phase, flow frequency characteristics, flow rate

## Abstract

Flow characteristic monitoring includes parameters such as flow regime, fluid characteristic frequency, and flow rate, which are crucial for optimizing production and ensuring the safety of oil and gas transportation systems. Existing fluid monitoring technologies, such as various flow meters, often face limitations in providing distributed and real-time monitoring data. In contrast, distributed acoustic sensing offers a spatial resolution of 1 m with high frequency sampling capability, allowing for long-term, multi-point dynamic monitoring of fluid migration characteristics. We developed an indoor physical simulation pipeline loop to assess the feasibility of using distributed acoustic sensing for monitoring flow migration characteristics. The experiment collected signal characteristics under different conditions, including background noise, single gas-phase flow, single liquid-phase flow, and gas–liquid two-phase flow. In the frequency–power spectral density analysis, single gas-phase flow signals are concentrated at lower frequencies, single liquid-phase flow displays noticeable spikes over a broader frequency range, and gas–liquid two-phase flow covers the widest frequency range with stronger amplitude signals. Autocorrelation analysis shows larger oscillations for gas–liquid two-phase flow, smoother signals for gas-phase flow, and more turbulent signals for liquid-phase flow. By examining root mean square energy changes, flow rates can be qualitatively estimated, revealing a positive correlation between energy and flow velocity. Finally, the study discussed the limitations of the experimental setup and proposed improvements and advanced research directions of distributed acoustic sensing in fluid monitoring.

## 1. Introduction

Oil and gas are essential primary energy sources that support contemporary industries and economies [1]. Gaining insight into the real-time production conditions within the wellbore during the extraction of oil, natural gas, and gas hydrates is crucial for maintaining operational safety and refining production strategies [2,3,4]. Among the various factors involved, the gas-to-liquid ratio stands out as particularly significant, as it directly affects the optimization of production processes. Timely adjustments to this ratio are necessary to improve both recovery efficiency and safety [5].

Multiphase Flow Meters (MPFMs) are commonly utilized in the oil and gas sector to measure the flow rates of various phases, such as oil, gas, and water. The precision of MPFMs is influenced by the measurement technologies they employ, including differential pressure, Coriolis, and capacitance sensors. While MPFMs are effective in delivering real-time data, their application in downhole environments faces significant challenges. These include the high cost of sensors, and the harsh conditions found deep underground, which complicate their deployment and reliability [6,7,8,9].

Differential pressure flowmeters operate by creating a pressure drop using an orifice plate or venturi to measure flow rates. While they perform well with clean liquids and gases, their accuracy can be compromised in multiphase flow conditions [6]. Positive displacement flowmeters, which rely on rotating or oval gears to calculate flow rates, offer high accuracy for clean fluids but are less effective with high-viscosity liquids and tend to be more expensive [7]. Magnetic flowmeters utilize Faraday’s law of electromagnetic induction to measure conductive fluids, delivering precise and reliable results for liquids like water, though they are not suitable for non-conductive fluids [10]. Ultrasonic flowmeters use the transit time of ultrasonic pulses between transducers to measure flow rates, making them effective for clean or dirty liquids, gases, and steam, and they can be measured in both directions [8]. Vortex flowmeters calculate flow rate by measuring the frequency of vortices created by a bluff body, which is ideal for clean gases and liquids but not for multiphase flows [11]. Coriolis flowmeters determine the mass flow rate by measuring the inertia forces induced by the flow, offering high accuracy for both liquids and gases, with bidirectional measurement capabilities. However, they tend to be more costly [12]. Thermal flowmeters estimate flow by evaluating heat transfer from a heated sensor to the fluid, making them suitable for gases, though their accuracy can be affected by fluid viscosity [6].

An alternative method involves tracer-based techniques, where chemical tracers are injected into the fluid flow, and their concentrations are measured to estimate the gas–liquid ratio [13]. These techniques provide valuable information about the flow regime and gas–liquid ratios by analyzing tracer distribution based on concentration measurements. However, their use is limited by the need for continuous tracer injection, which can be costly and impractical for long-term monitoring [14]. Additionally, managing traced materials to prevent environmental impacts can be challenging, and the process can be labor-intensive, making it unsuitable for frequent or ongoing use. The resolution of tracer monitoring is also constrained by the injection rate and sampling frequency.

Production Logging Tools (PLTs) are used to collect flow data along the wellbore by measuring parameters such as temperature, pressure, and flow rate. These tools provide valuable information about flow profiles. However, PLTs offer only a snapshot of the flow conditions during the logging process and do not support continuous monitoring, limiting their usefulness for real-time decision-making [15]. Additionally, PLTs require the well to be shut in or production to be halted during logging operations, which can disrupt production schedules and incur substantial costs. The accuracy of PLT measurements can also be influenced by wellbore conditions, such as scale accumulation or equipment fouling.

While these methods provide useful data, they are often limited by factors like high costs, challenges in real-time monitoring, and limited spatial resolution due to the well depth and harsh downhole environments. Direct measurement with traditional sensors is particularly challenging and expensive in deep wells, and such sensors are unable to deliver high-resolution monitoring in these conditions.

Distributed Acoustic Sensing (DAS) technology has emerged as a promising solution to these challenges. DAS utilizes fiber optic cables to deliver continuous, real-time data along the entire length of the monitored area. As illustrated in Table 1, existing flowmeters are effective for measuring flow at a single point. However, implementing long-term, real-time distributed monitoring with these flowmeters can be prohibitively expensive, and there is still significant potential for further development in industrial applications. In contrast, DAS can offer dynamic, high-frequency monitoring of gas–liquid two-phase flow at a much lower cost. DAS boasts several advantages, including high sensitivity, extensive spatial coverage, and the ability to detect flow dynamics without the need for intrusive sensors. This technology has proven especially valuable in characterizing complex flow behaviors, making it a promising tool for improving production efficiency and enhancing safety in oil and gas operations.

The capability of accurately characterizing fluid flow dynamics with spatial resolutions down to 1 m demonstrates the effectiveness of DAS technology, enabling detailed assessment of flow behavior throughout the pipeline [16]. Moreover, systems utilizing fiber optic cables possess inherent resistance to electromagnetic disturbances, thus proving particularly advantageous in environments subject to intense electromagnetic noise [17]. Optical fibers’ inherent durability further enhances the reliability of DAS systems, as their robust nature provides excellent resistance to environmental factors, ensuring stable performance over extended operational periods [18].

Additionally, fiber optic sensing technology enables effective monitoring over extensive pipeline distances, making it versatile for applications involving diverse fluids, such as oil, gas, and water [19]. The simple structure of fiber optic sensing solutions also facilitates ease of installation and upkeep, significantly lowering engineering expenses [20]. Furthermore, these systems operate without electromagnetic emissions, thereby ensuring enhanced safety [21]. DAS monitoring is particularly valuable in environments where traditional single-point sensors are not practical, such as downhole, high-temperature, high-pressure, and long-distance scenarios. Each point along the optical fiber serves as a sensing location, allowing the system to simultaneously monitor features and transmit signals.

This study employs DAS to monitor vibrations induced by fluid flow through pipelines, extracting key flow characteristics, such as flow frequency, fluid phase, and fluid composition, through the analysis of these vibration signals. The research lays the groundwork for using DAS in monitoring oil and gas production processes, offering valuable insights into the characterization of fluid and gas production profiles. These advancements will contribute to enhancing the understanding and optimization of oil and gas production operations.

The paper begins with a review of current fluid flow monitoring methods, discussing their principles and highlighting areas that require further improvement. The second section introduces the monitoring instruments, experimental setup, and procedures used in physical simulation experiments. The third section analyzes the results from fluid flow monitoring with DAS, providing an interpretation of the observed flow characteristics. The fourth section discusses the experimental phenomena and suggests improvements for future research. Finally, the paper concludes with a summary of the key findings.

## 2. Methods

### 2.1. Experimental Setup

#### 2.1.1. Mechanism of High-Definition Distributed Acoustic Sensing

**Figure 1:**
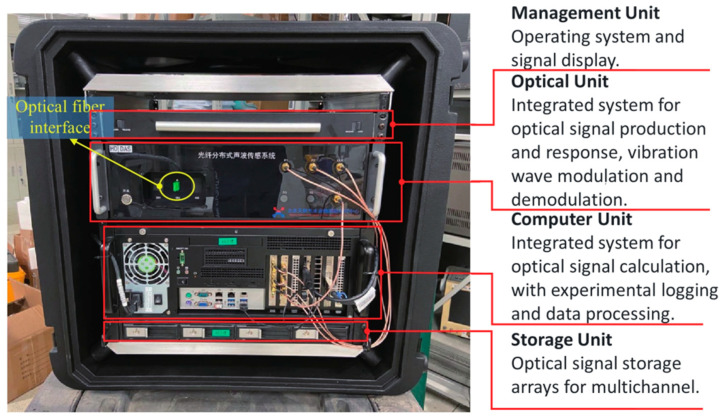
Component units and functions of HD-DAS.

DAS is a sensing technology that utilizes the optical properties of optical fibers to detect acoustic waves. High-Definition Distributed Acoustic Sensing (HD-DAS) is a state-of-the-art technique that uses a single optical fiber to continuously measure acoustic and real-time vibration signals across the fiber’s entire length. As illustrated in Figure 1, the method generates paired pulses via heterodyne-based frequency shift modulation, sends them through the optical fiber, and utilizes Rayleigh scattering for signal detection [16,22]. By acquiring data at 1 m intervals, HD-DAS delivers high-resolution and dependable monitoring in wellbores extending up to 2500 m. With a wide dynamic range spanning 0–2000 Hz and over 100 dB, along with a noise floor lower than −80 dB re rad/√Hz, it could continually monitor passive acoustic all day long without an external sound source.

In the Shenhu area of the South China Sea, HD-DAS was employed to monitor the production of natural gas hydrate [23]. By enabling observation of the entire wellbore in real-time, the system clearly differentiated distinct zones—such as the seawater section, a curved segment, and horizontal production intervals. Notably, HD-DAS detected a 52.12 Hz vibration from the electric submersible pump in the curved section, and the signal-to-noise ratio reached 27 dB. Additionally, it successfully tracked downhole tool movements, supported operational tasks, and used rate analysis of characteristic signals to identify various fluid types. These successful results highlight the potential of HD-DAS as a valuable method for all-encompassing acoustic monitoring in natural gas hydrate production.

HD-DAS operates as a dual-pulse heterodyne phase-sensitive Optical Time-Domain Reflectometer (Φ-OTDR), engineered for high-fidelity vibration measurement and waveform reconstruction. It launches two optical pulses at frequencies *f*1 and *f*2 into the fiber, separated by a time offset Δt. Those pulses generate Rayleigh backscatter, with vibrations along the fiber introducing phase shifts in the interference signal between them. Subsequently, a specialized heterodyne demodulation algorithm extracts these phase variations, making it possible to detect vibration events and reconstruct their waveforms with high precision.

In the HD-DAS system, the correlation linking phase variation φ (in radians) to strain change *τ* (in pε) is given by the following formula:

(1) $$\tau =\frac{10^{12}}{\frac{2\pi nL}{\lambda }\left[1+\frac{n^{2}}{2}\left(-P_{11}+2\mu P_{12}\right)\right]}\varphi$$

where *n* = 1.5 denotes the refractive index of the optical fiber, *L* = 7 m represents the gauge length, *λ* = 1550 nm indicates the wavelength, *P*_11_ = 0.121 and *P*_12_ = 0.270 are the elastic-optic coefficients, and *μ* = 0.17 corresponds to Poisson’s ratio. Therefore, the strain variation can be expressed in terms of the phase change as *τ* = 2.1 × 10^4^*φ* [23].

**Figure 2:**
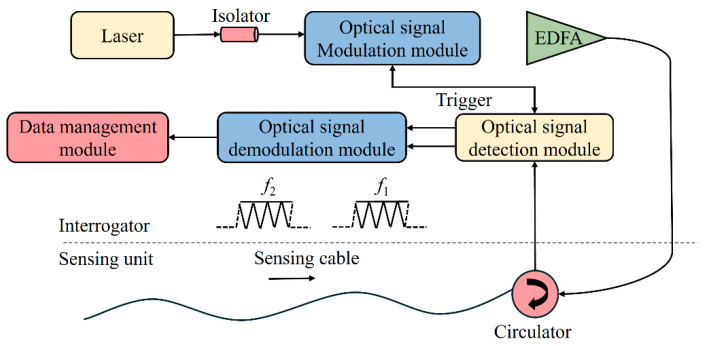
Diagram illustrating the structure of the HD-DAS system (Modified from [23]).

The HD-DAS system comprises several core modules [16,22,23,24]. As illustrated in Figure 2, an optical signal modulation unit generates heterodyne pulse pairs, which are then boosted by an erbium-doped fiber amplifier (EDFA). And a circulator guides these pulses into the sensing fiber and routes the backscattered signals toward the detection module, where a heterodyne demodulation algorithm extracts phase-change details from the returning optical signals. The sensing optical fiber itself captures acoustic signals, providing multiple distributed sensing channels throughout its entire length. Subsequently, a dedicated demodulation unit analyzes the phase changes, yielding highly accurate phase measurements. Finally, a data processing module records and evaluates these measurements to extract critical insights about acoustic occurrences. By unifying modulation, amplification, pulse injection, detection, and demodulation within a single integrated framework, the system facilitates practical, real-time analysis and characterization of acoustic events.

#### 2.1.2. Experimental Loop

**Figure 3:**
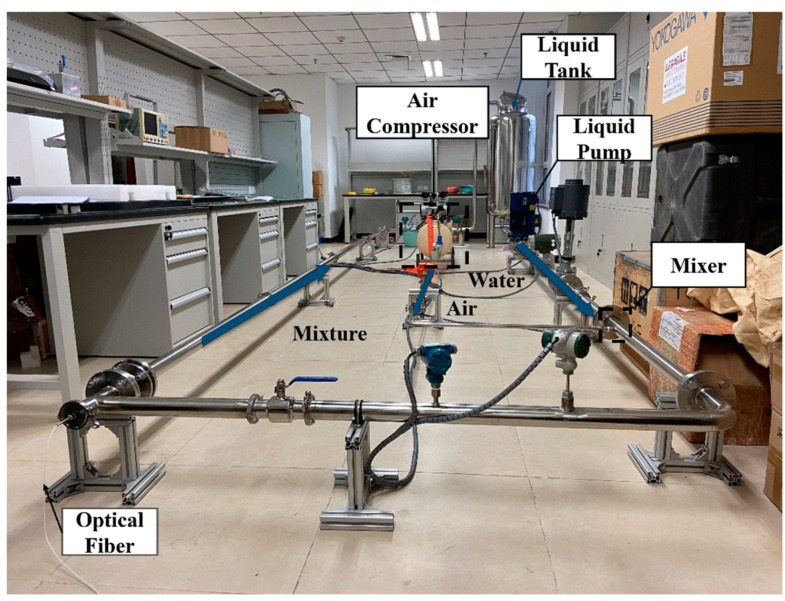
Air–water circulation device.

Figure 3 depicts the experimental setup designed to study gas–liquid flow behaviors. The system comprises distinct subsystems for air flow generation, liquid circulation, and their mixing in a mixer. For the liquid subsystem, water is kept in a water tank and circulated by a pump running at 35 Hz. An adjustable control valve maintains precise regulation of the flow rate. After completing the flow loop, the water returns to the reservoir for reuse. A thermometer continuously monitors the system temperature, ensuring stability at 25 °C throughout the experiments.

On the gas side, ambient air is compressed and stored in an air tank. A pressure-reducing valve dictates outlet flow, while a gas valve fine-tunes the airflow to achieve desired conditions. Because the gas phase mainly consists of air, it is simply vented after traversing the monitoring section, requiring a continuous supply to sustain flow conditions.

A fiber optic cable is placed centrally along the pipeline, and senses vibrations generated by the air–water flow, closely replicating deployment approaches used in the production of natural gas hydrate. The arrangement of this fiber optic cable is consistent with actual field installations. Within the simulation loop, a specialized control unit manages the flow rates, while the HD-DAS system continuously records vibration and optical signals in real time.

**Figure 4:**
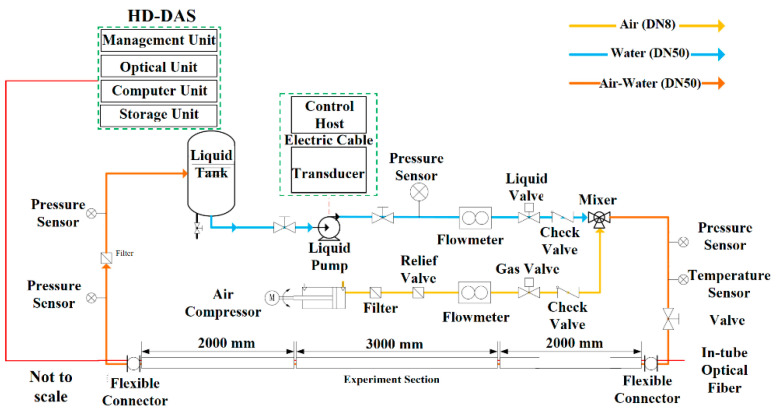
Diagram of the gas–liquid two-phase flow simulation experiment setup.

Figure 4 shows the experimental setup used to investigate gas–liquid two-phase flow behavior. The test pipe has an inner diameter of 50 mm. A liquid pump draws water from a tank and delivers it to the mixer. Flow adjustment on the liquid side is handled by a control valve, with a check valve ensuring fluid moves only in the intended direction. On the gas side, a pressure-reducing valve governs air pressure, and a gas control valve regulates airflow. A second check valve confirms proper air flow toward the mixer, where the gas merges with the liquid phase.

After exiting the mixer, gas and water flow through sensors measuring temperature and pressure to ensure accurate flow monitoring. Subsequently, it flows into a specialized pipeline equipped with a fiber optic sensing system designed to capture the dynamic properties of the fluid. These sensor data are continuously transmitted in real-time to the HD-DAS system, which conducts modulation and demodulation of signals, facilitating comprehensive analysis of flow characteristics. A fiber optic cable positioned centrally within the pipeline incorporates a reflective element at its distal end to minimize Fresnel reflections. The fiber’s proximal end connects directly to the HD-DAS unit, completing the optical pathway necessary for precise detection and characterization of the two-phase flow.

### 2.2. Experiment Process

The study established three experimental cases, including liquid-only and gas–liquid flow conditions. Detailed flow rates used in these experiments are provided in Table 2.

Before the formal experiment, we first used DAS to collect background noise in a quiet state, recording 20 sets of data, each lasting 10 seconds. The quiet state refers to the stationary state of the fluid inside the pipe. When the fluid remains stationary, regardless of whether the pipe contains water or not, there are no significant differences in the signals collected by the HD-DAS device. Recording background noise serves as a baseline for subsequent single-phase and two-phase experiments, aiming to eliminate the influence of the experimental environment on the results.

In the single gas-phase test, we maintained a constant pressure of 0.3 MPa using the relief valve. The gas flow rate was controlled by adjusting the gas valve opening to 5%, 7%, and 9% and measured by a mass flow meter. After opening the gas valve, we continuously recorded ten sets of data, each lasting ten seconds.

In the single liquid-phase experiment, we introduced only liquid into the experimental pipeline, setting the liquid pump frequency to 30 Hz. In the liquid flow experiment, due to the experimental setup’s maximum flow velocity being less than 1.2 m/s and the Reynolds number being under 2000, only laminar flow filling the pipe was observed during the experiment. The liquid flow valve was adjusted in increments, starting at 20% and increasing by 20% per step up to 100%. A mass flow meter was used to measure the actual flow rate. For each flow rate, we continuously recorded 10 sets of data, each lasting 10 s.

Finally, in the gas–liquid flow experiment, both gas and liquid were introduced into the experimental pipeline simultaneously. The liquid pump was set to operate at 30 Hz, and the relief valve pressure was maintained at 0.3 MPa. Flow variation was achieved by adjusting both gas and liquid valves. Specifically, we set the gas valve to 5% and 10% and the liquid valve to 20% and 60%, resulting in four experimental groups for DAS monitoring. Each group underwent ten repeated measurements, with each measurement lasting 10 s [16].

### 2.3. Data Analysis Methods

#### 2.3.1. Root Mean Square

The calculation of the root-mean-square (RMS) in the context of DAS involves estimating the relative average-strain amplitude for each fiber-optic channel. The RMS is calculated for each channel using the following formula:

(2) $$\mathrm{RMS}=\sqrt{\frac{1}{n}\sum_{i=1}^{n}x_{i}^{2}}$$

where $x_{i}$ is the strain amplitude of the *i*-th sample in an *n*-sample-long time series. This approach helps estimate the amplitude of the strain signals recorded by the fiber-optic cable during different operational stages, such as single-phase and multiphase flow. By using the RMS values, we assess the variations in strain transmitted from the fluid flow, providing insights into the coupling properties between the optic signals and fluid flow characteristics [25].

#### 2.3.2. Power Spectral Density

Power spectral density (PSD) is a method for describing how the power of a signal or time series is distributed over different frequency components. In signal processing, PSD is an important tool for analyzing the frequency content of signals, providing insights into their energy distribution and identifying dominant frequency components.

Welch’s method is an improved non-parametric approach for estimating the power spectral density (PSD) of a signal, designed to reduce the variance of the spectral estimate and improve the stability of the periodogram. The method achieves this by segmenting the signal, computing the average spectrum of each segment, and thereby smoothing the spectral estimate and reducing the effect of noise [26,27].

First, assume the input signal *x*[*n*] has a length of N. The signal is divided into several overlapping segments, each with a length of *M*, with an overlap of *L* (typically L < M) between adjacent segments. The segmented signals are

*x_k_*[*n*], *k* = 0, 1, …, *K* − 1 (3)

where *K* is the number of segments, calculated as

(4) $$K=\text{⌊}\frac{N-L}{M-L}\text{⌋}$$

where ⌊.⌋ represents the floor function.

Then, each segment is multiplied by a window function to reduce the edge effects on the frequency spectrum estimation. Common window functions include Hanning and Hamming windows. Let *w*[*n*] represent the window function, and the windowed signal is

*X_k_*[*n*]*w*[*n*], *n* = 0,1,…,*M −* 1 (5)

In addition, the discrete Fourier transform is performed on each windowed signal to obtain the spectrum for each segment:

(6) $$X_{k}[f]=\sum_{n=0}^{M-1}x_{k}\left[n\right]w\left[n\right]e^{-j2\pi fn/M}$$

where $X_{k}$[*f*] is the frequency domain representation (i.e., spectrum) of the *k*-th segment at frequency *f*.

The power spectral density is calculated by

(7) $$P_{k}[f]=\frac{1}{U}{\left|X_{k}[f]\right|}^{2}$$

where *U* is the normalization factor for the window energy, defined as

(8) $$U=\frac{1}{M}\sum_{n=0}^{M-1}{\left|w\left[n\right]\right|}^{2}$$

This ensures that the energy of the windowed signal does not become distorted due to the window function.

Finally, average the PSDs of all segments to obtain the Welch PSD estimate:

(9) $$P_{xx}[f]=\frac{1}{K}\sum_{k=0}^{K-1}P_{k}\left[f\right]$$

#### 2.3.3. Autocorrelation Function

The autocorrelation coefficient is a statistical measure used to quantify the correlation between different time points or spatial locations in time series or spatial data. The value of the autocorrelation coefficient reflects the continuity or periodicity of a sequence in time or space, thus, revealing the inherent patterns in the data [28,29,30].

Let the time series be *X =* [*x_1_,x_2_,…,x_T_*], where *T* is the length of the time series. The autocorrelation coefficient *r_k_* describes the correlation of the sequence at a time lag of *k*, and can be expressed as

(10) $$r_{k}=\frac{\sum_{t=1}^{T-k}(x_{t}-\bar{x})\left(x_{t+k}-\bar{x}\right)}{\sum_{t=1}^{T-k}{\left(x_{t}-\bar{x}\right)}^{2}}$$

where *k* is the lag order, which represents the time interval for examining the autocorrelation of the sequence, $x_{t}$ is the *t*-th value in the sequence, and $\bar{x}$ is the mean of the time series.

## 3. Experimental Results and Analysis

### 3.1. Flow Phase Identification

**Figure 5:**
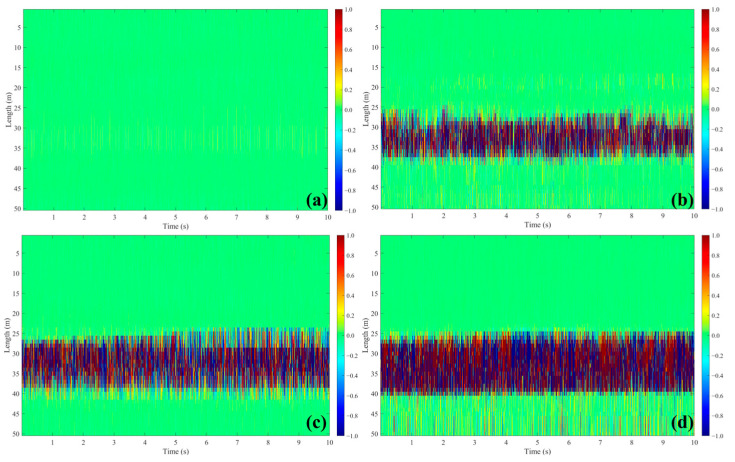
DAS waterfall plots of four flow phases: (**a**) background noise, (**b**) single gas phase, (**c**) single liquid phase, and (**d**) gas–liquid two-phase flow.

The waterfall plots depicted in Figure 5 provide a comprehensive visualization of the fluid flow vibration signals in the pipe recorded by the HD-DAS system under various fluid phases. Analyzing these plots, it becomes apparent that the gradient of the fluid flow characteristics, derived from the DAS-captured vibration signals, can be effectively utilized to measure the flow types with a high degree of accuracy.

In this study, the DAS system successfully recorded single gas flow, single water flow, and gas–water two-phase flow, which agree with the predetermined pipeline flow phase. This correlation validates the reliability and precision of the DAS system in monitoring and measuring pipeline flow.

Furthermore, the waterfall plot provides an intuitive method for qualitatively identifying the flow rate. The intensity of the vibration signals on the waterfall plot effectively indicates the relative flow rate, enabling real-time monitoring of fluid vibration characteristics within the pipeline.

By analyzing the waterfall plots of different flow phases, we observed that the pipeline has a signal collection and amplification effect. The background noise signals detected by the optical fiber inside the pipeline still exhibit weak signals, which are stronger than those in a free state. Moreover, the introduction of gas and liquid into the pipeline significantly increased the signal intensity detected by DAS. Among the different flow phases, the gas–liquid two-phase flow has the most vigorous signal intensity, followed by the single liquid phase, with the single gas phase showing the weakest signal intensity. Moreover, after introducing gas and liquid into the pipeline, the signal intensity was significantly higher than the background noise. The waterfall plot effectively identified the starting times of gas and liquid production.

**Figure 6:**
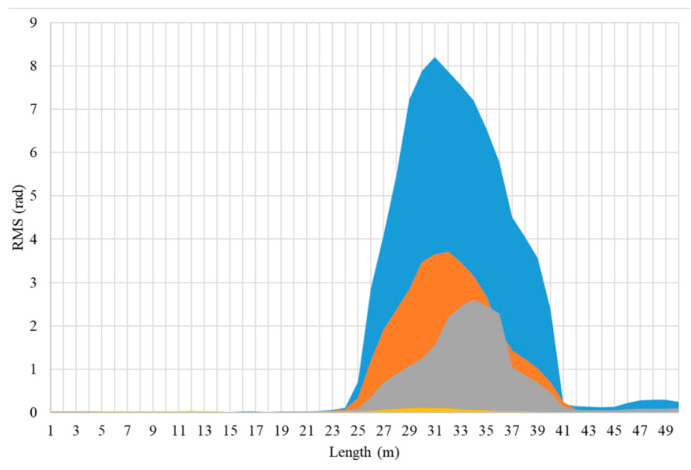
Plot of RMS calculation results. The blue box, orange box, gray box, and yellow box represent gas–liquid two-phase flow, single-phase liquid flow, single-phase gas flow, and background noise, respectively.

Figure 6 presents the RMS calculation results across different channels for the four flow phases. The blue block represents gas–liquid two-phase flow, the orange block indicates single liquid-phase flow, the gray block represents single gas-phase flow, and the yellow block denotes background noise. The vertical axis corresponds to the RMS values, while the horizontal axis represents the fiber signal channels. Due to the equipment spatial sampling interval being 1 m and the gauge length set at 7 m, the signal range exceeds the actual experimental pipeline length by 7 m. The results in the figure also confirm the observations from the waterfall plot analysis: the gas–liquid two-phase flow exhibits the highest signal intensity, followed by the single liquid-phase flow, and the single gas-phase flow is the weakest. Nevertheless, introducing fluid into the pipeline increased the signal intensity detected by the fiber optic sensor.

In Figure 6, from the numerical results, the RMS maximum values for gas–liquid two-phase flow, single-phase liquid flow, and single-phase gas flow are 8.2, 3.6, and 2.5, respectively. Additionally, across the various channels in the experimental pipe, the energy relationship, from highest to lowest, is as follows: gas–liquid two-phase flow, single-phase liquid flow, and single-phase gas flow.

**Figure 7:**
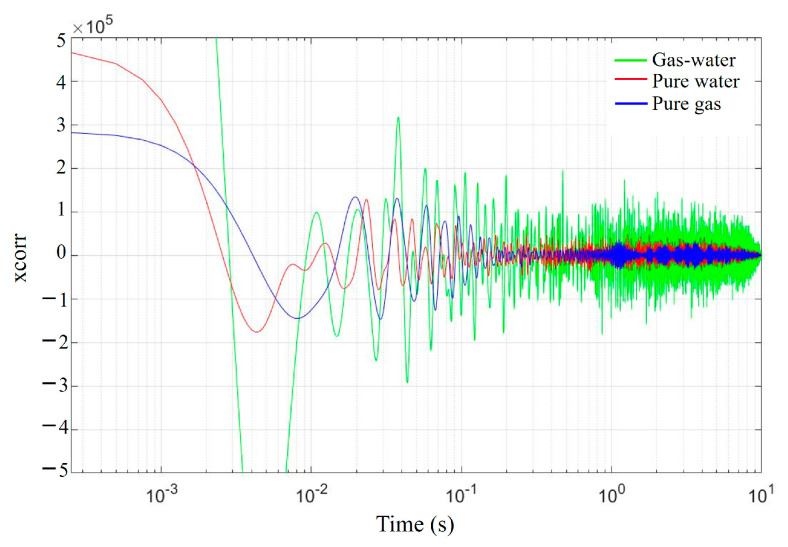
Autocorrelation coefficient plot for three flow phases.

Different flow phases exhibit significant differences and distinct trends by analyzing the autocorrelation coefficient of the vibration signals generated during the fluid flowing through the pipeline, as shown in Figure 7. In Figure 7, the green line, red line, and blue line represent the autocorrelation coefficient variations in gas–liquid two-phase flow, single liquid-phase flow, and single gas-phase flow, respectively. The autocorrelation of pure gas vibration signals is relatively smooth, showing regular oscillations. In contrast, the autocorrelation of pure water vibration signals is more chaotic, with reduced regularity. The autocorrelation of gas–water two-phase flow vibration signals features denser oscillations with moderate regularity.

In summary, we utilized the waterfall plot to directly observe the energy color-bar and calculated and analyzed the RMS values of different channels. These analyses provided methods to characterize and differentiate the fluid features of three flow phases and background noise.

### 3.2. Investigation of Fluid Characteristic Frequencies

**Figure 8:**
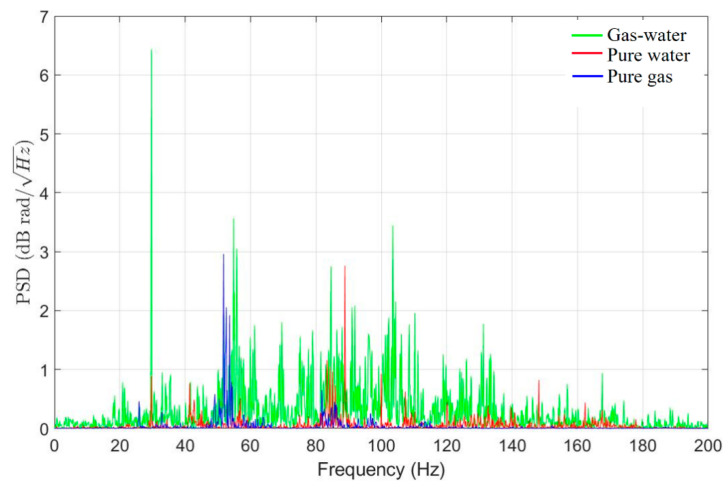
Frequency-PSD plot for three flow phases.

Furthermore, based on Figure 6, we selected channel 32 with the highest signal intensity and focused on the significant signal frequency range of 0–200 Hz for further analysis. In Figure 8, the green line, red line, and blue line represent the PSD results as a function of frequency for gas–liquid two-phase flow, single liquid-phase flow, and single gas-phase flow, respectively, within the 0–200 Hz range. By examining the characteristic frequency bands of gas–liquid two-phase flow, single gas-phase flow, and single liquid-phase flow in the frequency domain, we found that the spectrum of pure gas vibration signals is relatively concentrated and features lower frequencies. The pure water vibration signals have distinct sharp spectral lines, covering a broader frequency range. The gas–water two-phase flow vibration signals exhibit significant spectral enhancement, incorporating characteristics of both pure gas and pure water.

**Figure 9:**
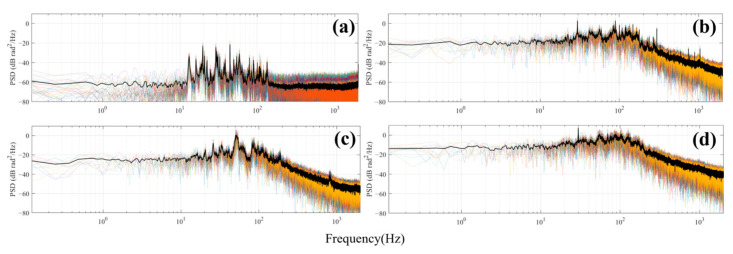
Frequency-PSD plot: (**a**) background noise, (**b**) single liquid phase, (**c**) single gas phase, and (**d**) gas–liquid two-phase flow.

Figure 9 is based on Figure 8, with the horizontal axis of frequency transformed logarithmically, while retaining the original values for the vertical axis. This data compression into a more manageable range facilitates more straightforward observation and comparison, allowing for a more precise comparison of characteristics across different frequency intervals.

Figure 9 presents the signal characteristics of channel 32 under four conditions: background noise, pure water, pure gas, and gas–water two-phase flow. Each figure shows the results of 10 records, with the black curve in the PSD representing the average of these 10 records. The figure shows that the background noise spectrum is primarily concentrated in the 13 Hz to 134 Hz range and exhibits discrete spectral characteristics, which may result from environmental noise resonating within the pipeline. The pure water condition shows a distinct spectral line at 30 Hz, while the pure gas spectrum is much smoother, lacking sharp spectral lines. The air–water flow also shows a prominent spectral line at 30 Hz.

### 3.3. Analysis of Flow Rate Features

**Figure 10:**
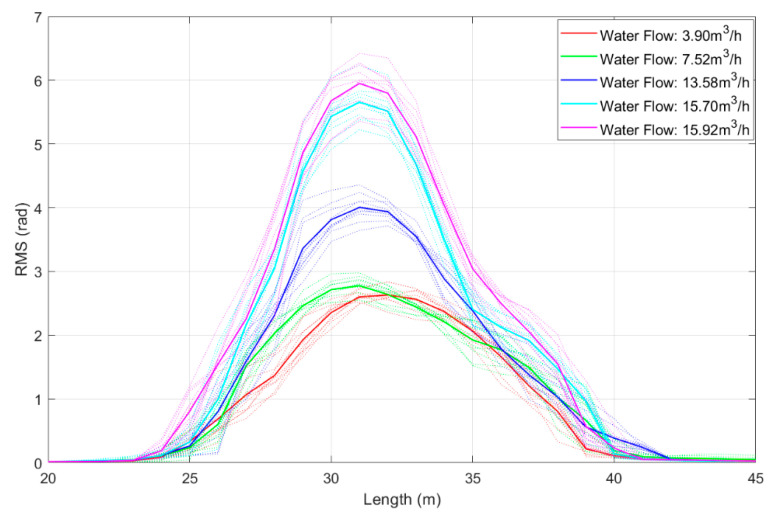
Plot of RMS results for each channel in the experimental pipeline with only liquid introduced. Besides, the dashed line represents the result of a single experiment, and the solid line represents the average value of ten groups of data.

Based on the RMS calculation method mentioned in Section 2, we calculated the RMS values for each channel in the experimental pipeline with only liquid introduced. Subsequently, we averaged the results from 10 recordings for each data set, as shown in Figure 10. In this figure, different colored curves represent corresponding flow rates, as indicated in the legend. Additionally, the light-colored lines represent the individual monitoring results from each of the 10 measurements, while the dark-colored lines represent the averaged values.

In addition, for the RMS results of each channel, there is a proportional relationship between energy and flow rate. The RMS energy increases approximately as the liquid flow rate increases. Moreover, it is evident that after averaging the data, both the stability and reliability of the results have been further improved. These experimental findings provide a feasible approach for further exploring the relationship between fluid energy and flow rate.

**Figure 11:**
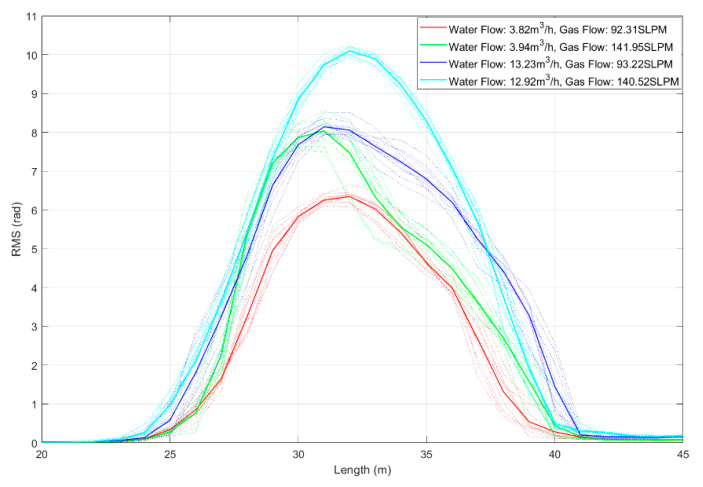
Plot of RMS results for each channel in the experimental pipeline with gas–liquid two-phase flow introduced. Besides, the dashed line represents the result of a single experiment, and the solid line represents the average value of ten groups of data.

To further analyze the energy characteristics of the vibration signals, we also calculated the RMS values for each channel under different gas and liquid flow rates. The results, averaging over 10 recordings for each flow combination, are presented in Figure 11. In this figure, different colored curves correspond to various flow rates, as shown in the legend. The light-colored lines depict the individual results from the 10 measurements, while the dark-colored lines indicate the averaged values.

For the RMS values under different flow combinations, an increase in gas flow results in higher vibration signal energy when the liquid flow rate remains constant. Similarly, with a continuous gas flow rate, increasing the liquid flow also leads to increased signal energy. These results indicate that both liquid and gas flow rates contribute to an increase in signal amplitude, suggesting a proportional relationship between flow rate and energy. However, it is essential to note that multiple solutions can exist regarding the relationship between energy and flow rate. For instance, the energy levels of the green and blue lines are similar, but they represent different liquid and gas flow rates.

**Figure 12:**
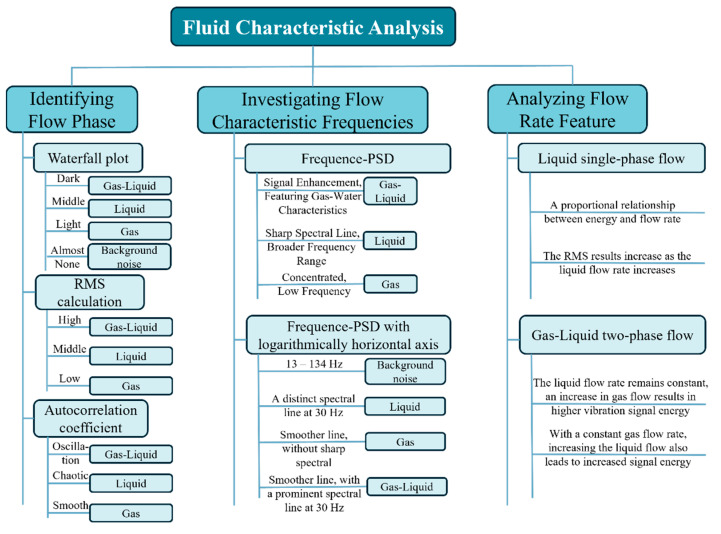
Summary plot of fluid characteristics analysis.

In summary, we conducted a comprehensive analysis of fluid characteristics based on the content in Section 3, as illustrated in Figure 12 and Table 3. First, we identified different flow phases; in the waterfall plot, the dark plot represents gas–liquid two-phase flow, the medium color represents single liquid-phase flow, the lighter color indicates single gas-phase flow, and the almost signal-free areas correspond to background noise. Furthermore, we analyzed fluid characteristic frequencies. The background noise spectrum is mainly concentrated in the 13 Hz to 134 Hz range, the pure gas spectrum is relatively smooth without sharp spectral lines, and both the pure liquid phase and gas–liquid two-phase show distinct spectral lines at 30 Hz, with the pure liquid phase showing sharper lines. Additionally, we analyzed the flow velocity characteristics, and it is evident that increasing the flow rate of both the liquid and gas phases leads to an enhanced vibration signal.

## 4. Discussion

We used the HD-DAS system to monitor fluid characteristics in the pipeline, verifying that it can be utilized to investigate pipeline flow phases, characteristic frequencies, and flow rates.

This experiment effectively distinguished the phase states of different fluids using methods such as waterfall plots, RMS calculation results, and autocorrelation coefficients. However, while we identified the fluid phases, we could not differentiate the flow regimes effectively. For future experiments, we propose using transparent pipelines for visual observation of flow regimes and applying flow regime calculations to categorize the various flow rate combinations observed in the experiments. This approach will also allow us to investigate the relationship between characteristic frequencies and flow phases.

As the flow rate increasing of any phase leads to a rise in flow energy, it is necessary to accurately determine the flow phase before quantitatively assessing the flow velocity. In our research, we propose effectively distinguishing flow phases by examining the waterfall plot color-bar values, the fluid flow spectrograms, and accumulated RMS results. In further studies, wavelet transform, and other advanced signal processing methods can be adopted to enhance flow-phase discrimination.

Additionally, this experiment analyzed the PSD energy at different frequencies to determine the characteristic flow frequencies for three different flow phases. However, due to the limitations in flow range and larger intervals between experimental groups, we cannot confirm that the obtained fluid flow characteristic frequencies represent a universal pattern for this pipeline flow phase. For future investigations, we propose expanding the experimental range, employing more effective filtering methods, and altering the pipeline material to identify characteristic frequencies for different flow regimes accurately. Furthermore, integrating numerical simulation methods using commercial software such as COMSOL Multiphysics and Fluent could provide deeper insights into the fluid flow characteristic frequencies.

The experiment qualitatively explored the positive correlation between energy and flow rate by comparing the average RMS values at different flow rates, finding that an increase in flow rate leads to higher RMS values. However, in gas–liquid two-phase flow, both increasing gas and liquid flow rates contribute to enhanced vibration signals, resulting in multiple solutions regarding energy combinations and RMS values—different gas–liquid flow rate combinations can yield similar RMS values. Therefore, in field experiments, it may not be possible to determine which phase’s flow rate significantly enhances energy solely based on the magnitude of the RMS value. In further experiments, we can establish different models to separately measure and predict the gas–liquid two-phase flow rates using a controlled variable approach.

In addition, due to the limited number of experimental groups, this work could not establish a quantitative model for predicting the flow velocity of gas and liquid, and accurate values for gas and liquid flow rates could not be obtained. In further experiments, we suggest conducting tests with smaller gas–liquid flow rate intervals to improve the accuracy of the analysis. For single liquid-phase flow and gas–liquid two-phase flow, an increase in fluid flow velocity leads to changes in the amplitude of the generated vibration signals. The characteristic vibration frequencies vary with different flow rate combinations in the gas–liquid two-phase flow. Therefore, it is feasible to consider establishing a flow rate analysis and prediction model based on energy and characteristic frequency perspectives.

Furthermore, this experiment is a pilot study using the HD-DAS system to investigate the fluid migration characteristics of two-phase flow in indoor pipelines, validating the feasibility of DAS for monitoring fluid migration. The study found that DAS can detect fluid migration patterns in terms of energy and frequency. Therefore, in subsequent experiments, we can establish empirical formulas based on energy and characteristic mapping relationships based on frequency, enabling the measuring efficiency of flow rates and flow regimes.

In the actual operating conditions of liquid production wells, there are two types of production environments: the acoustically active section influenced by the working frequency of the water pump and the acoustically silent section unaffected by the pump frequency. In this experiment, due to pipeline scale limitations, the pump frequency affects the determination of liquid characteristic frequencies. Therefore, this experiment simulates the acoustic active section influenced by the pump’s operating frequency. For future experiments, we recommend placing the liquid pump at a location farther from the pipeline to simulate the acoustically silent section, which is not affected by the pump’s operating frequency. This approach will help identify the overall fluid migration patterns in production well.

There still exist problems in analyzing acoustic data captured by optical fiber in complex flow conditions. However, this technology holds great potential for providing non-invasive, real-time, and dynamic measurements [31]. With advancements in fiber optic monitoring technology, the resolution and gauge length of the HD-DAS system are expected to improve, enabling more accurate and detailed exploration of fluid migration characteristics. This will provide enhanced safety and operational efficiency for the oil and gas industry.

## 5. Conclusions

We utilized the HD-DAS system to monitor the fluid migration in indoor pipelines, capturing the vibration signals generated by fluid movement. Analyzing these signals, we derived conclusions about flow phases, fluid characteristic frequencies, and flow velocities. The results indicate that the HD-DAS system can accurately distinguish between gas-phase, liquid-phase, and gas–liquid two-phase flows. Additionally, using RMS values and autocorrelation coefficients, we were able to differentiate these flow phases. In the frequency-PSD figures, we identified characteristic frequencies corresponding to each flow phase. The vibration signal of a single gas-phase flow is more concentrated and mainly appears at lower frequencies. A single liquid-phase flow, however, shows noticeable spikes spanning a broader frequency range. Meanwhile, a gas–liquid two-phase flow exhibits the widest frequency range and stronger amplitude signals, covering both single gas-phase and single liquid-phase flow ranges. In the autocorrelation coefficient analysis, the gas–liquid two-phase flow shows larger oscillation amplitude, the single gas-phase flow appears smoother, and the single liquid-phase flow is more turbulent. In the flow rate analysis, we qualitatively determined the respective flow rates of single liquid-phase and gas–liquid two-phase flows based on RMS energy changes. We observed a positive correlation between fluid migration energy and flow velocity, where energy increases as the flow rate rises. These findings provide indicative suggestions for determining on-site oil and gas production conditions and relative flow rates of fluids.

## Figures and Tables

**Table 1:** Comparison table of application principle, advantages, and disadvantages of different flow meters.

Types of Flow Meters	Application Principle	Advantages	Disadvantages
Differential pressure flowmeters	Pressure difference	Suitable for various fluids, simple design, established technology	Pressure loss, sensitive to flow profile, inaccurate with viscous fluids
Positive displacement flowmeters	Use rotating or oval gears	Suitable for viscous fluids, independent of fluid properties	Limited flow range, sensitive to particulate matter
Magnetic flowmeters	Faraday’s law of electromagnetic	No moving parts, ideal for dirty or corrosive fluids	Unsuitable for non-conductive fluids, high initial cost
Ultrasonic flowmeters	Use the transit time of ultrasonic pulses	Non-intrusive, no pressure loss, no internal parts wear, strong adaptability	Requirement for clean fluids, affected by bubbles and solid particles
Vortex flowmeters	Measure the frequency of vortices	Good for high temperature and high-pressure applications, insensitive to fluid density and viscosity	Sensitivity to flow disturbances, limited to medium to high flow rates
Coriolis flowmeters	Measure the inertia forces	Direct mass flow measurement, no need for temperature or pressure compensation	Sensitive to vibration and external forces, power consumption
Thermal flowmeters	Evaluate heat transfer from a heated sensor	Applicable to gases and liquids, fast response time	Sensitivity to environmental conditions
Distributed acoustic sensing	Rayleigh scattering	Long-distance monitoring, real-time data, no electromagnetic interference, cost-effective for large areas	Requires skilled signal processing methods, cost of initial setup

**Table 2:** Gas and liquid flow rates for single and multiphase experiments.

Single-Phase Gas Flow *Q*_g_ (LSPM)				
91.04		117.94		142.27
Single-phase liquid flow *Q*_l_ (m^3^/h)				
3.90	7.52	13.78	15.70	15.92
Gas and liquid two-phase flow				
*Q*_l_ (m^3^/h)			*Q*_g_ (LSPM)	
3.82			92.31	
13.23			93.22	
3.94			141.95	
12.92			140.52	

**Table 3:** Summary table of experimental results.

	Types	Flow Characteristics
Flow phases	Gas–liquid	Dark waterfall plot High RMS
	Liquid	Middle waterfall plot Middle RMS
	Gas	Light waterfall plot Low RMS
	Background noise	Almost none
Flow characteristic frequencies	Gas–liquid	Signal enhancement, a spectral line at 30 Hz
	Liquid	Sharp spectral line, broader frequency
	Gas	Concentrated, low frequency
	Background noise	13–134 Hz
Flow rate features	Liquid single-phase flow	The RMS results increase as the liquid flow rate increases
	Gas–liquid two-phase flow	As the flow rate of any phase increases, the fluid flow energy increases

## Data Availability

The original contributions presented in this study are included in the article. Further inquiries can be directed to the corresponding author.
